# The Role of Family and Peer Influence in Shaping Teen Smoking Attitudes in Romania

**DOI:** 10.3390/jcm15051819

**Published:** 2026-02-27

**Authors:** Ana-Luiza Iorga, Ioana Munteanu, Maria Beatrice Catrangiu, Andrei Șerban Zanfirescu, Mihai Octavian Dan, Florin-Dumitru Mihălțan, Dragoș Băiceanu, Antonela Dragomir, Simona Pârvu, Ioana-Mădălina Moşteanu, Alexandra Paraschiv, Raluca Bobocea, Mara Amalia Bălteanu, Oana-Andreea Parliţeanu, Daniel Radu, Alina Croitoru, Viorel Jinga, Beatrice Mahler

**Affiliations:** 1 “Marius Nasta” Institute of Pneumology, 050159 Bucharest, Romania; ana-luiza.iorga@drd.umfcd.ro (A.-L.I.); ioana.munteanu2015@yahoo.ro (I.M.); maria-beatrice.catrangiu@rez.umfcd.ro (M.B.C.); dragos.baiceanu@marius-nasta.ro (D.B.); madalina.mosteanu@yahoo.com (I.-M.M.); alexandra.paraschiv08@yahoo.com (A.P.); mara.balteanu@prof.utm.ro (M.A.B.); alina.haulinca@umfcd.ro (A.C.); beatrice.mahler@umfcd.ro (B.M.); 2 Faculty of Medicine, Titu Maiorescu University, 040051 Bucharest, Romania; radudaniel301979@gmail.com; 3 Department of Psychology and Cognitive Science, Faculty of Psychology and Educational Sciences, 050663 Bucharest, Romania; serban.zanfirescu@unibuc.ro; 4 Faculty of Medicine, “Carol Davila” University of Medicine and Pharmacy, 050474 Bucharest, Romania; mihai-octavian.dan0720@stud.umfcd.ro (M.O.D.); florin.mihaltan@umfcd.ro (F.-D.M.); simona.parvu@umfcd.ro (S.P.); raluca.bobocea@marius-nasta.ro (R.B.); viorel.jinga@umfcd.ro (V.J.); 5 European Network for Smoking and Tobacco Prevention, 1050 Brussels, Belgium; 6 National Institute of Public Health, 050463 Bucharest, Romania; 7 Doctoral School, University of Medicine and Pharmacy Craiova, 200349 Craiova, Romania; 8 Ilfov County Emergency Clinical Hospital, 022104 Bucharest, Romania

**Keywords:** smoking, tobacco use, smoking identities, teenagers, smoking-associated risks, social perspectives

## Abstract

**Background**: Despite a decline observed in smoking rates amongst adults in many high-income countries, tobacco usage among adolescents remains a significant concern, particularly in Central and Eastern Europe, Romania being no exception. This cross-sectional study aims to assess the prevalence of smoking among teenagers enrolled in several public schools in Bucharest and the surrounding county, Ilfov, while also exploring their attitudes, perceptions, and experiences regarding tobacco. **Methods**: A public health campaign, conducted in collaboration with multiple institutions between September 2023 and March 2024, sought to provide secondary school students with updated information on smoking and its implications through presentations at schools. Following these presentations, students voluntarily completed anonymous on-paper questionnaires consisting of 10 multiple-choice questions designed to gauge their knowledge and attitudes towards smoking, conceptually inspired by internationally validated instruments such as the Global Youth Tobacco Survey. **Results**: A total of 945 teenagers participated in our study, with a median age of 13.04 years (standard deviation of ±1.08). Results indicate that 22.85% (n = 216) of teenagers had attempted smoking at the time of the investigation. Additionally, 57.88% (n = 547) of respondents reported exposure to second-hand smoke at home, and 40.42% (n = 382) had been invited to smoke previously. **Conclusions**: Various factors influence smoking behaviors among teenagers, with peer pressure and familial background playing significant roles in shaping their attitudes toward smoking. Our study highlights the vulnerability of the young Romanian population to these influences, emphasizing the need for initiatives aimed at mitigating tobacco use and fostering a healthier future environment. Nonetheless, these findings shall serve as an instrument for the development of school-based prevention programs and stricter tobacco usage policies.

## 1. Introduction

Smoking is one of the leading risk factors for preventable diseases, particularly affecting cardiovascular and respiratory health. It has also been linked to mental disorders such as major depressive disorder, anxiety, and suicide attempts [1]. Today, smoking remains a global public health threat, with over 8 million deaths annually attributed to tobacco use, including those caused by second-hand smoke exposure. Although smoking prevalence remains high worldwide, developing countries report a greater number of smokers compared to higher-income nations [2].

In Romania, which has only recently been classified as a developed country by the World Bank, the Global Adult Tobacco Survey—a project supported by the World Health Organization (WHO)—indicates that in 2018, 30.7% of Romanian adults were classified as active smokers, a notable increase from 26.8% in 2011 [3]. The relatively permissive legislative framework regarding smoking, coupled with extensive marketing campaigns, contributes to an environment that appears accepting of smokers. Over the past few decades, Romania has implemented measures such as banning smoking in enclosed public spaces (Law no. 15/2016) and requiring graphic health warnings on tobacco packaging to address this issue [4,5,6]. However, public focus has increasingly shifted toward heated tobacco products, which have been heavily marketed in recent years and remain exempt from indoor smoking bans [7,8].

According to the U.S. Centers for Disease Control and Prevention (CDC), nearly 90% of current adult smokers first tried smoking during their teenage years, making this demographic a critical target for anti-smoking initiatives. For instance, in the United States, 12.6% of high school students currently use tobacco products [9]. Teenagers are particularly susceptible to peer influence and aggressive marketing strategies, necessitating further government action to foster a healthier society. Adolescents are at a developmental stage characterized by heightened sensitivity to social acceptance and belonging, making them more vulnerable to peer pressure.

Social cognitive theories, such as Bandura’s Social Learning Theory, underscore the influence of peers in shaping adolescent smoking behaviors. The appeal of smoking is often intensified by its portrayal in media and marketing aimed at youth, which resonates with adolescents’ desires for autonomy and identity formation. Additionally, factors such as personality traits, coping strategies, and emotional regulation significantly influence adolescent smoking behavior. For example, individuals with lower self-esteem or higher impulsivity levels may turn to smoking as a coping mechanism for stress or emotional regulation. Furthermore, adverse childhood experiences, such as parental substance use or exposure to trauma, can heighten the likelihood of engaging in risky behaviors like smoking during adolescence [10].

Peer smoking status, alongside peer group norms, emerges as a consistent predictor of adolescent smoking attempts’ initiation, as well as smoking behavior modeling, a feature reported by multiple studies. For instance, a 2017 meta-analysis comprising 75 studies revealed a nearly double likelihood of initiating and continuing smoking in adolescents with smoking friends [11]. Peer networks further explain the way adolescents influence and are influenced by entourage in smoking behaviors [12]. Additionally, parental smoking and family environment may contribute to adolescent smoking risk, yet evidence suggests a rather indirect influence than direct imitation of parent behaviors [13,14].

This cross-sectional study aims to assess the attitudes of Romanian teenagers aged 11–16 toward smoking and explore how their backgrounds influence their social beliefs on the topic. Additionally, it seeks to determine the prevalence of both first-hand and second-hand smoking within this age group.

## 2. Materials and Methods

Study Design: A public health campaign aimed at providing children aged 11–16 with updated information about smoking and its health risks was conducted by the “Marius Nasta” Institute of Pneumology in partnership with several organizations, including the “Carol Davila” University of Medicine and Pharmacy, the Romanian Ministry of Education, the Faculty of Psychology and Education Sciences at the University of Bucharest, the European Network for Smoking and Tobacco Prevention, the National Institute of Public Health, the Association for Education in Respiratory Disease, and the Medical Students’ Society of Bucharest. The campaign took place from September 2023 to March 2024, targeting 70 secondary schools in Bucharest and the surrounding County Ilfov. Teams of doctors, psychologists, and medical students visited each school to deliver interactive presentations designed to educate students about the risks associated with smoking. Following these presentations, students voluntarily completed anonymous on-paper questionnaires consisting of 10 multiple-choice questions aimed at assessing their social knowledge and attitudes toward smoking. The questionnaire was inspired by internationally validated instruments such as the Global Youth Tobacco Survey—GYTS [15]. However, it does not represent a validated adaptation of any existing standardized tool. The rationale of utilizing this questionnaire was to adapt the survey’s content to scholars’ level of understanding, as well as to respond to the original campaign objectives. The complete structure of the questionnaire is available in Appendix A.

Inclusion criteria: The study included only students physically present at the presentations held in schools, whose parents/guardians had previously signed an informed consent agreement in order for project enrollment. All completed questionnaires were subsequently included for data analysis.

Data retrieval and analysis: Responses were collected from the on-paper forms distributed to students in ten participating schools. The data were subsequently digitized and organized in a Microsoft Excel spreadsheet database, with no exclusion criteria applied during data collection. Statistical analysis was conducted using Microsoft Excel version 16.66.1 (2022 Microsoft). The chi-squared test (two-tailed) was used for quantitative variables, and a *p* value of <0.05 was considered statistically significant. Given the exploratory nature of the study, coupled with the use of a non-validated questionnaire, the analysis was limited to descriptive, univariate methods.

## 3. Results

### 3.1. Cohort Demographics

A total of 945 teenagers voluntarily participated in this cross-sectional study, with a median age of 13.04 years (standard deviation of ±1.08 years). Among the participants, 49.31% were female (n = 466), 44.97% were male (n = 425), and 54 respondents preferred not/omitted to disclose their gender.

### 3.2. Responders’ Background Regarding Smoking

The majority (57.88%, n = 547) of respondents reported living with an active smoker at the time of questionnaire completion. Additionally, 40.42% (n = 382) of teenagers indicated having been invited to try smoking in the past, with a slightly higher proportion among those living with an active smoker (46.07%, n = 252 versus 32.66%, n = 130 amongst children not exposed to smoking at home, *p* < 0.05). Regarding smoking attempts, 22.85% (n = 216) of respondents admitted to having tried smoking, showing minimal, not statistically significant variation by gender (20.00%, n = 85 among males vs. 23.60%, n = 110 among females, *p* = 0.22).

**Figure 1:**
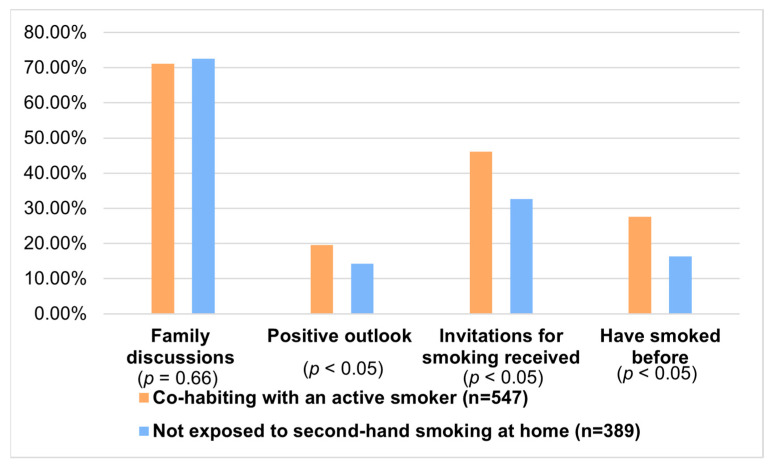
Comparisons regarding family discussions, perspective about smoking, and previous smoking experience between the two subgroups in terms of second-hand smoking exposure at home. The complete data are available in the appendix of this paper.

Family discussions about smoking behaviors were reported by 71.85% (n = 679) of respondents, with little difference between those living with active smokers (71.11%, n = 389) and those in smoker-free homes (72.61%, n = 289) (*p*-value = 0.66). Notably, 13.75% (n = 130) believed their families would support them if they decided to smoke, with higher percentages among those who had discussed the topic with their families (14.13%, n = 96 vs. 12.78%, n = 34, *p* = 0.66) and those living with active smokers (16.45%, n = 90 vs. 9.82%, n = 39, *p* = 0.004). Figure 1 presents a comparison between the subgroup cohabiting with an active smoker and children not exposed to second-hand smoking at home, regarding family discussions, smoking perception, and previous experience with smoking.

### 3.3. Perceptions and Attitudes Towards Smoking

Most of the respondents (85.29%, n = 806) reported feeling uncomfortable in a smoking environment, with 22.85% (n = 216) stating they were completely bothered by second-hand smoke. Some differences were noted between those living with smokers and those in smoker-free homes; 18.28% (n = 100) of the former group expressed a positive or neutral feeling in a smoking environment, compared to only 9.82% (n = 39) in the latter group, *p* < 0.05.

Additionally, 17.35% (n = 164) of respondents believed that smoking positively influences others’ perceptions of a person, with a slightly higher rate among those living in smoker households (19.56%, n = 107 compared to 14.32%, n = 57, *p* = 0.04) and those who discussed smoking with their families (19.14%, n = 130 vs. 12.78%, n = 34, *p* = 0.02). However, only 12.27% (n = 116) thought that smoking behaviors enhance one’s self-esteem.

**Figure 2:**
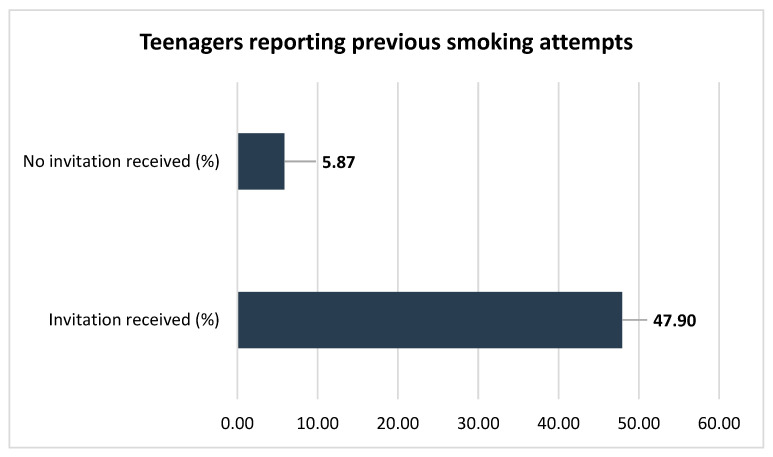
Comparison of smoking attempts between teenagers invited and not invited to smoke (*p* < 0.05).

Peer pressure emerges as a critical factor in teenagers’ attempts to smoke, with 47.91% (n = 183) responding positively to smoking invitations, compared to only 5.86% (n = 33) of their counterparts who denied having been invited to smoke (Figure 2), *p* < 0.05. This observation showcases first-time smoking attempts not as purely individual pursuits, but rather as socially influenced first tries.

**Figure 3:**
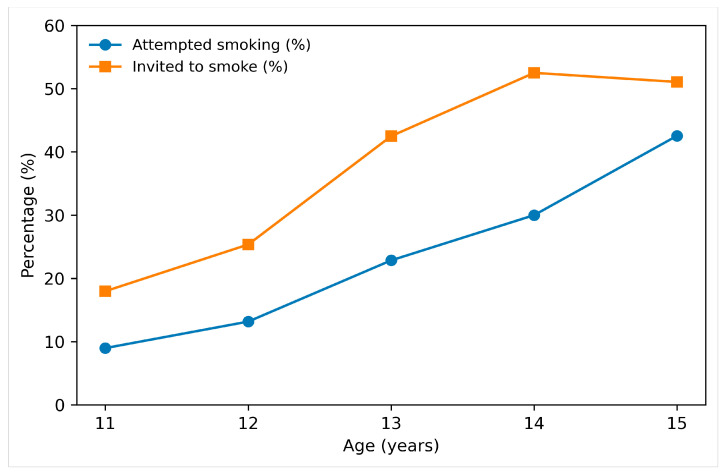
Diagram showing the ascending trend in smoking invites and attempts among teenagers in our cohort.

Upon dividing our results by age group, a clear connection emerged between older teenagers and smoking attempts. While multiple factors contribute to this trend, teenagers reporting peer invitations were more likely to respond positively to smoking attempts. A concerning finding, however, is that 17.97% of 11-year-olds have already received invitations to smoke, with the figure rising dramatically to 52.50% among 14-year-olds. A visual representation of the patterns of smoking invitations and attempts across age groups can be found in Figure 3.

A summary of information retrieved from the questionnaires and a more comprehensive comparison of results by gender, exposure to second-hand smoking at home, and partaking in family discussions are available in the appendix of this paper (Appendix B, Table A2 and Table A3).

## 4. Discussion

Various factors shape individuals’ perceptions of smoking behaviors and their implications, particularly among teenagers. Our study indicates that, while there is little variation in smoking behaviors between genders, factors such as the presence of an active smoker in the household and open discussions about smoking within the family significantly influence adolescents’ perceptions. These findings align with existing literature on the topic [10,16,17,18].

Similar to findings from other studies, the overall prevalence of smoking attempts among teenagers in our cohort was notably high at 22.85% (n = 216), with a slightly higher incidence among female respondents [16,19]. However, this is significantly lower than those reported in studies focusing on lower-income populations, suggesting that living conditions and median income may also influence smoking patterns among different groups [20].

Legislative measures at the government level are essential for discouraging smoking, particularly among younger populations, along with mitigating the healthcare burdens associated with this widespread phenomenon [21,22]. Research indicates that interventions such as increasing tobacco taxes can significantly reduce smoking-related hospitalizations, especially for respiratory diseases. However, effective population-level smoking reduction efforts require collaboration among governmental authorities, healthcare professionals, patient associations, and non-governmental organizations. These partnerships can enhance the impact of public health campaigns aimed at targeted populations [23].

It is crucial to examine how a legislative framework addressing heated tobacco products can be established in Romania. These products have garnered significant interest among younger individuals, who are susceptible to extensive marketing campaigns that emphasize appealing product characteristics, such as various flavors [24,25,26]. Furthermore, the current legal framework permits the use of heated tobacco products indoors, creating a regulatory gap that must be addressed by restrictive measures, such as the complete ban of indoor use or the creation of designated heated tobacco smoking areas.

Several theoretical frameworks help explain smoking behavior. Bandura’s Social Learning Theory posits that smoking behavior can be learned through observing significant others, suggesting that attitudes and values related to smoking are partly shaped by these observations [27]. Consistent with this theory, previous studies have identified parental, sibling, and peer smoking as significant risk factors for adolescent smoking uptake. Additionally, Self-Determination Theory suggests that behavior is influenced by the fulfillment of three basic psychological needs: autonomy, competence, and relatedness [28]. For instance, individuals who feel pressured to smoke by social norms may experience conflict with their need for autonomy, thereby impacting their smoking behavior [29].

The chemical substances in tobacco promote an inflammatory process that can severely exacerbate conditions such as community-acquired pneumonia. Community models implemented in other countries illustrate the importance of tobacco-free policies enacted by decision-makers [30].

Future research should include more comprehensive longitudinal studies, employing multi-center designs in order to provide further descriptions and correlations between psychosocial and environmental determinants of smoker identities among Romanian adolescents. Peer influence, family habits, and novel marketing and social media campaigns shall represent the central focus in order to facilitate designing evidence-based prevention strategies, along with targeted public health interventions [31,32].

To address this issue effectively, compliance with existing regulations must prioritize limiting access to tobacco, especially in public spaces, alongside educational initiatives supported by the Romanian government. Tobacco regulations should be continuously updated to align with emerging needs, consistent with WHO guidelines for tobacco control [33].

Limitations: This study presents several limitations that should be acknowledged. Firstly, although the study included a large number of participants, this sample size is only a small fraction of school-aged children in the Bucharest Metropolitan Area, which may lead to limited generalizability on a population level. Moreover, the study included only school-enrolled adolescents, while it is well-reported that smoking rates tend to be higher in adolescents not attending educational forms, thus leading to a possible underestimation of actual figures. Additionally, our sample consists solely of students from metropolitan areas; participating schools may thus not be a valid representation of Romanian scholars as a whole. Research indicates that non-metropolitan adolescents are more likely than their metropolitan counterparts to report lifetime smoking, initiate smoking at younger ages, and experience greater acceptance of smoking behaviors from friends and parents, as highlighted by a 2018 study [34]. Moreover, we have used an adapted, non-internationally validated questionnaire, which may limit reproducibility, alongside the absence of multivariate modeling. A final limitation to consider is the reliance on self-reported data, which is susceptible to bias, as the findings are based entirely on the responses of the participating teenagers, which may be influenced by the honesty of their answers. Despite the challenges in generalizability due to potential selection bias and sampling limitations, cross-sectional studies like this one can serve as valuable starting points for further research.

## 5. Conclusions

Smoking is a phenomenon characterized by considerable variability among populations, yet background factors, social influences, and exposure play critical roles in shaping teenagers’ smoking behaviors. This study suggests that the young population in Romania is particularly susceptible to these influences, with family and environmental factors significantly impacting adolescents’ perceptions and behaviors regarding smoking. Interventions aimed at adolescents should enhance their decision-making skills, coping strategies, and resistance to peer pressure. Many adolescents perceive smoking as a means of asserting independence or gaining acceptance within peer groups, highlighting the need for interventions that address the underlying psychological motivations for smoking initiation. Educational programs should not only inform about the health risks of smoking but also equip adolescents with the skills necessary to resist social pressures and make informed choices regarding tobacco use.

## Data Availability

The data presented in this study are available upon reasonable request from the corresponding author.

## Appendix A

Complete questionnaire structure

**Table A1:** Questionnaire structure.

Question Number	Question	Possible Answer 1	Possible Answer 2	Possible Answer 3
1	What is your age? ^1^			
2	What is your gender?	Male	Female	I prefer not to declare
3	How do you feel in a place where people smoke? ^2^	Pleasant	Unpleasant	Strongly bothered by the smoke inhaled
4	Do you live with a smoker?	Yes	No	-
5	Have you talked about smoking with your family?	Yes	No	-
6	Do you think your family would support you if you become a smoker?	Yes	No	-
7	Do you think smoking positively influences other people’s perception of you?	Yes	No	-
8	Does smoking improve self-esteem?	Yes	No	-
9	Have you ever been invited to smoke?	Yes	No	-
10	Have you ever tried smoking?	Yes	No	-

^1^ First question is an open-answer question. ^2^ Question no.3 allows more than one answer.

## Appendix B

**Table A2:** Summary of answers retrieved from the questionnaire.

Total Cohort: N = 945 (100%)			
Feature	Answer 1—% (n=)	Answer 2—% (n=)	Answer 3—% (n=)
Gender	Male—44.97% (n = 425)	Female—49.31% (n = 466)	Prefer not to mention—5.71% (n = 54)
How they feel in a smoking environment	Pleasant—14.70% (n = 139)	Unpleasant—85.29%, (n = 806)	Completely deranged by the smoke inhaled—22.85% (n = 216)
Co-inhabitants with a smoker	Yes—57.88% (n = 547)	No—42.11% (n = 398)	Not answered—0.001% (n = 1)
Discussions in the family regarding smoking	Yes—71.85% (n = 679)	No—28.14% (n = 266)	
Family support if taking up smoking	Yes—13.75% (n = 130)	No—86.24% (n = 815)	
Smoking positively influences others’ outlook on a person	Yes—17.35 (n = 164)	No—82.64% (n = 781)	
Smoking enhances a person’s self esteem	Yes—12.27% (n = 116)	No—87.51% (n = 827)	Not answered—0.002% (n = 2)
Invited to smoke	Yes—40.42% (n = 382)	No—59.47% (n = 562)	Not answered—0.001% (n = 1)
Attempted smoking	Yes—22.85% (n = 216)	No—77.03% (n = 728)	Not answered—0.001% (n = 1)

**Table A3:** Complete comparison of answers among gender, second-hand smoking at home, and teenagers who took part in family discussions regarding smoking.

Feature	Men n = 425 44.97%	Women n = 466 49.31%	Exposed to Second-Hand Smoking at Home n = 547 57.88%	Not Exposed to Second-Hand Smoking at Home n = 398 42.11%	Took Part in Family Discussions Regarding Smoking n = 679 71.85%	Have not Taken Part in Family Discussions Regarding Smoking n = 266 28.14%
How they feel in a smoking environment	Pleasant—9.41%, n = 40 Unpleasant—90.58%, n = 385 Completely deranged by the smoke inhaled—24.70%, n = 105	Pleasant—17.38%, n = 81 Unpleasant—82.61%, n = 385 Completely deranged by the smoke inhaled—22.53%, n = 105	Pleasant—18.28%, n = 100 Unpleasant—81.71%, n = 447 Completely deranged by the smoke inhaled—19.56%, n = 107	Pleasant—9.79%, n = 39 Unpleasant—90.20%, n = 359 Completely deranged by the smoke inhaled—27.13%, n = 108	Pleasant—15.75%, n = 107 Unpleasant—84.24%, n = 572 Completely deranged by the smoke inhaled—23.26%, n = 158	Pleasant—12.03%, n = 32 Unpleasant—87.96%, n = 234 Completely deranged by the smoke inhaled—21.42%, n = 57
Have discussed the subject with the family	Yes—68.47%, n = 291 No—31.52%, n = 134	Yes—75.32%, n = 351 No—24.67%, n = 115	Yes—71.11%, n = 389 No—28.88%, n = 158	Yes—72.61%, n = 289 No—27.38%, n = 109	X	X
Family support if taking up smoking	Yes—11.76%, n = 50 No—88.23%, n = 375	Yes—14.59%, n = 68 No—85.4%, n = 398	Yes—16.45%, n = 90 No—83.54%, n = 457	Yes—9.79%, n = 39 No—90.20%, n = 359	Yes—14.13%, n = 96 No—85.86%, n = 583	Yes—12.78%, n = 34 No—87.21%, n = 232
Smoking positively influences others’ outlook on a person	Yes—17.41%, n = 74 No—82.58%, n = 351	Yes—17.59%, n = 82 No—82.40%, n = 384	Yes—19.56%, n = 107 No—80.43%, n = 440	Yes—14.32%, n = 57 No—85.67%, n = 341	Yes—19.14%, n = 130 No—80.85%, n = 549	Yes—12.78%, n = 34 No—87.21%, n = 232
Smoking enhances a person’s self esteem	Yes—9.88%, n = 42 No—89.88%, n = 382	Yes—13.3%, n = 62 No—86.48%, n = 403	Yes—14.99%, n = 82 No—84.82%, n = 464	Yes—8.54%, n = 34 No—91.20%, n = 363	Yes—12.81%, n = 87 No—87.18%, n = 592	Yes—10.90%, n = 29 No—88.34%, n = 235
Invited smoking	Yes—34.58%, n = 147 No—65.17%, n = 277	Yes—43.56%, n = 203 No—56.43%, n = 263	Yes—46.06%, n = 252 No—53.93%, n = 295	Yes—32.66%, n = 130 No—67.33%, n = 268	Yes—42.56%, n = 289 No—57.43%, n = 390	Yes—34.96%, n = 93 No—64.66%, n = 172
Attempted smoking	Yes—20.00%, n = 85 No—79.76%, n = 339	Yes—23.60%, n = 110 No—76.39%, n = 356	Yes—27.60%, n = 151 No—72.39%, n = 396	Yes—16.33%, n = 65 No—83.41%, n = 332	Yes—24.30%, n = 165 No—75.69%, n = 514	Yes—19.17%, n = 51 No—80.45%, n = 214

Note: Lack of answer was also taken into account when calculating percentages.
